# Double trouble: methanol outbreak in the wake of the COVID-19 pandemic in Iran—a cross-sectional assessment

**DOI:** 10.1186/s13054-020-03140-w

**Published:** 2020-07-09

**Authors:** Hossein Hassanian-Moghaddam, Nasim Zamani, Ali-Asghar Kolahi, Rebecca McDonald, Knut Erik Hovda

**Affiliations:** 1 Social Determinants of Health Research Center, Shahid Beheshti University of Medical Sciences, Tehran, Iran; 2 Department of Clinical Toxicology, Loghman Hakim Hospital, School of Medicine, Shahid Beheshti University of Medical Sciences, South Karegar Street, Tehran, Iran; 3 National Addiction Centre, Institute of Psychiatry, Psychology and Neuroscience, King’s College London, London, UK; 4 The Norwegian CBRNE Centre of Medicine, Department of Acute Medicine, Oslo University Hospital, Oslo, Norway

**Keywords:** Alcohol, Ethanol, Poisoning, Mortality, Coronavirus

Iran has been the epicenter of COVID-19 in the Middle East, with a total of 120,198 infected cases and 8556 deaths as of June 10 [1]. The pandemic has been complicated by the co-occurrence of a large methanol outbreak in Iran, seemingly triggered by false claims that consumption of disinfectants and alcohols could prevent and treat COVID-19 infection. According to local news, the ensuing rise in ethanol demand made bootleggers decolorate industrial alcohols containing pyridine (to deter from consumption) using bleach, before selling them as regular ethanol to Iranians.

In this research letter, we describe the scale of the Iranian methanol outbreak, based on hospitalization and mortality data collated from databases of the Iranian Ministry of Health (MOH) and Legal Medicine Organization (LMO) for the period of February 23 (first documented COVID-19 case in Iran) until May 2, 2020. MOH records indicate 5876 hospitalizations for methanol poisoning, occurring in geographical clusters, with just three (Tehran, Fars, Khuzestan) out of the total 31 Iranian provinces accounting for the majority (52.2%; *n* = 3068) of cases (see Table 1). In terms of mortality, MOH reported that 534 patients with methanol poisoning were confirmed dead in the hospital setting, equivalent to an estimated case fatality rate of approximately 9% (534/5876). LMO registered 800 deaths from methanol poisoning during the same period (see Table 1 and Fig. 1), comprising both in-hospital and community-based fatalities. This 33% discrepancy in deaths between MOH and LMO data (i.e., (800–534)/800 × 100) may have several explanations. For instance, LMO data also includes out-of-hospital deaths and is likely more accurate. Moreover, a hospital-based diagnosis of methanol poisoning is difficult and complicated by the lack of diagnostic equipment or physician knowledge. Therefore, methanol poisoning may go undetected in hospitals, and accurate diagnosis is—best case scenario—only assigned during a post-mortem examination, which then enters LMO statistics. In the absence of formate analyses, late presenters can become false negatives.

**Table 1:** Methanol poisoning cases and fatalities in Iran (23 February to 2 May 2020)

Province	Poisoning cases: hospital admissions (source: MOH)	Methanol deaths*	
		In hospital (source: MOH)	Total registered (source: LMO)
Tehran	1177	87	205
Khuzestan	1079	93	88
Fars	812	99	139
Razavi Khorasan	581	67	78
East Azerbaijan	483	50	75
Alborz	248	43	52
Ardebil	223	22	31
Isfahan	207	6	19
Kerman	139	0	2
Kermanshah	132	2	2
Mazandaran	100	10	28
Yazd	96	12	10
Markazi	87	4	4
Kurdestan	79	0	9
The other provinces	433	39	58
**Total**	**5876**	**534**	**800**

*Brain-dead cases considered dead

*MOH* Ministry of Health, *LMO* Legal Medicine Organization (data is available through https://bit.ly/2WUBfZo)

**Fig. 1:**
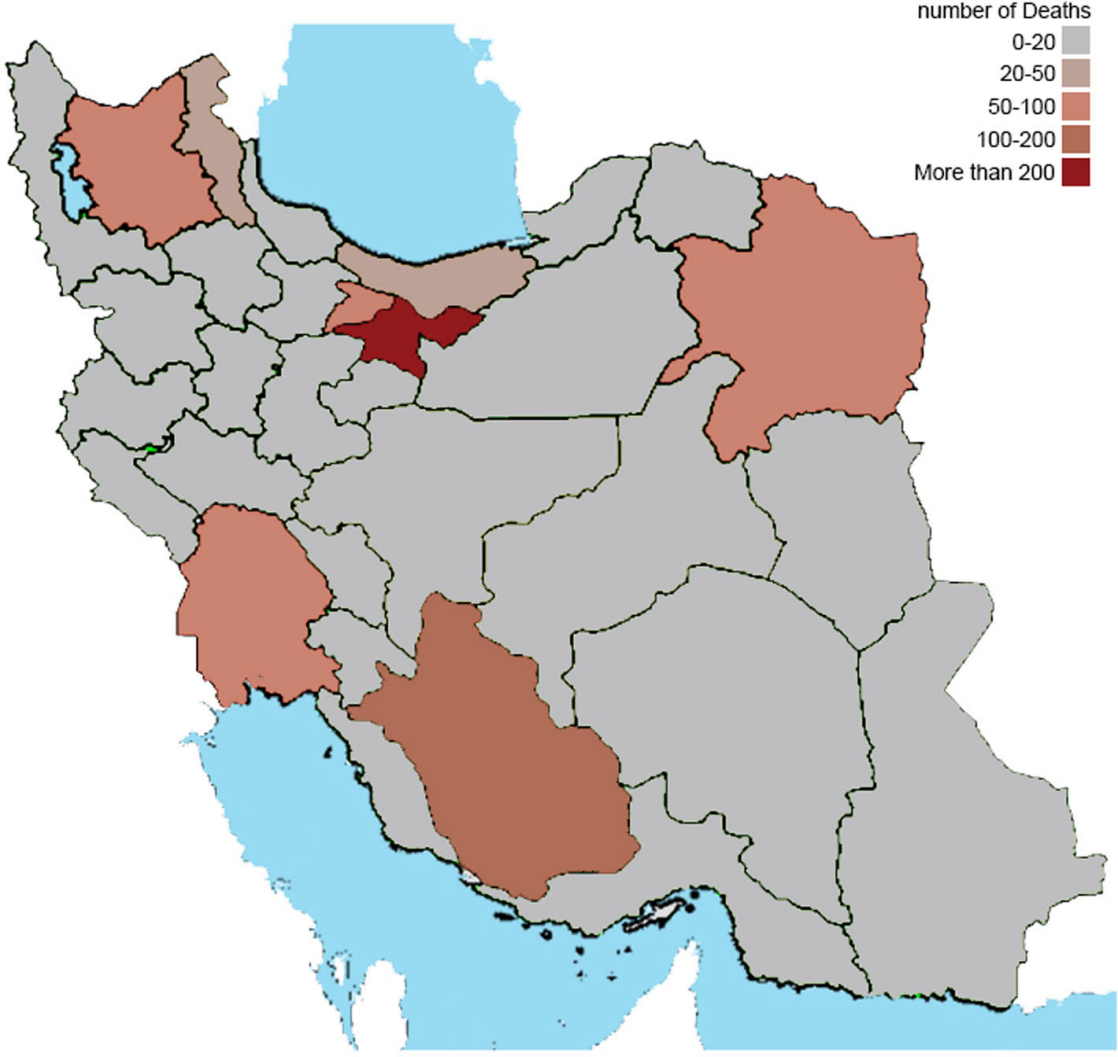
Mortality by province based on LMO data

Despite these inconsistencies, the number of Iranian poisoning cases (5876 hospitalizations from late February until early May),\ is already five times higher than the second-largest methanol outbreak in history, which was recorded in Libya in March 2013 and affected 1066 patients [2].

From our data, it is unclear how many Iranians drank adulterated alcohol for recreational purposes or as gastrointestinal “disinfectant” to prevent—or treat—COVID-19 infection. With no end to the COVID-19 pandemic in sight, it is thus paramount to educate the general public that alcohol does not protect against COVID-19, as already initiated by the WHO [3]. The United Nations rightly recognizes the international spread of “fake news” related to COVID-19 as a threat to human lives [4], as health care systems have to contend with medical misinformation of the general public.

In methanol poisoning, the efficacy of treatment is markedly reduced in delayed presentations. With 534 methanol deaths registered by MOH and 800 deaths by LMO, alongside 5876 hospitalizations, we report an estimated mortality rate in the range of 9–14%. However, this figure is preliminary and should be interpreted with great caution, until LMO and MOH release their final data. Additionally, in many more non-fatal methanol poisoning cases, adults and children are now vision-impaired or blind from this toxic alcohol.

During situations like pandemics, healthcare institutions may seem dangerous settings, which could pose a risk of viral infection. It is possible that fear of COVID-19 kept methanol poisoning victims from seeking and obtaining timely care. This highlights the importance of early identification and initiation of treatment, which can be supplemented by “active case finding” by treating physicians and public health agencies [5]. Public health messaging and strategic planning are crucial to prevent future methanol outbreaks [6].

## Data Availability

All the data is presented in the text.
